# The effects of L-Citrulline and Glutathione on Endurance performance in young adult trained males

**DOI:** 10.1080/15502783.2023.2206386

**Published:** 2023-04-26

**Authors:** Hannah E. Cabre, Casey E. Greenwalt, Lacey M. Gould, Abbie E. Smith-Ryan

**Affiliations:** a University of North Carolina at Chapel Hill, Applied Physiology Laboratory, Department of Exercise and Sport Science, Chapel Hill, NC; b University of North Carolina at Chapel Hill, Human Movement Science Curriculum, Department of Allied Health Science, Chapel Hill, NC

**Keywords:** Blood flow, vasodilation, critical velocity, running performance, dietary supplement

## Abstract

**Background:**

Citrulline may amplify the effects of L-arginine and nitric oxide concentration, which may augment vasodilation and blood flow, thereby enhancing aerobic exercise performance. The purpose of this randomized, double-blind, placebo-controlled crossover study was to investigate effects of L-citrulline + Glutathione on aerobic exercise performance and blood flow in well-trained men.

**Methods:**

Twenty-five males (Mean ± SD; Age: 22.2 ± 2.4 yrs; Height: 177.0 ± 4.8 cm; Weight: 75.3 ± 6.9 kg) were randomly assigned to the L-citrulline + Glutathione (Setria Performance Blend: SPB; L-citrulline [2 g] + glutathione [200 mg], 6 capsules) or placebo (PL; 3.1 g cellulose, 6 capsules) group. Participants performed a maximal oxygen consumption treadmill test to determine peak velocity (PV) and returned after eight days of ingesting either PL or SPB. Three timed treadmill runs to exhaustion (TTE) were performed at 90%, 100%, and 110% PV. Brachial artery blood flow and vessel diameter were assessed using ultrasound at 1-hr prior to exercise (1hrPrEX), after each exercise bout, immediately post-exercise (immediate PEX), and 30 minutes post exercise (30minPEX) at visits 2 and 4. Blood analytes were assessed via venous blood draws at visit 1, visit 3, and 1hrPEX, immediate PEX, and 30minPEX at visits 2 and 4. After a 14-day washout, participants repeated the same procedures, ingesting the opposite treatment. Separate repeated measures ANOVAs were performed for TTE, vessel diameter, blood flow, and blood analytes.

**Results:**

Blood flow was significantly augmented 30minPEX (*p* = 0.04) with SPB in comparison with PL. L-citrulline and L-arginine plasma concentrations were significantly elevated immediately PEX (*p* = 0.001) and 30-minPEX (*p* = 0.001) following SPB in comparison to PL.

**Conclusion:**

Acute ingestion of SPB after eight days may enhance blood flow, L-citrulline, and L-arginine plasma concentrations after high-intensity exercise, which may enhance performance.

**Clinical Trial Registration:**

[https://clinicaltrials.gov/ct2/show/nct04090138], identifier [NCT04090138].

## 1. Introduction

As the global sports nutrition market totaled $10.7 billion in 2020 with a projected growth of 10.9% from 2021 to 2028 [1], evaluations of sport nutrition ingredients are needed. Citrulline is growing in popularity for its potential benefits for blood flow and exercise performance [2]. Due to the performance implications of increased blood flow, L-citrulline is among one of the most common ingredients found in commercially available multi-ingredient pre-workout supplements, with 71% of pre-workout supplements including L-citrulline [3]. Understanding L-citrulline’s individual potential performance benefits is an important step in determining ideal timing, dosing strategies, and potential synergistic ingredients for co-ingestion.

During exercise, the demand for oxygen delivery and energy substrates is elevated in the working skeletal muscle. These demands are met with an increase in vasodilation and blood flow [4]. Citrulline may contribute to vasodilation via amplified nitric oxide (NO) concentration, which is most notably recognized as a potent stimulator of vasodilation [5]. Citrulline is efficiently recycled into L-arginine for subsequent NO production through the citrulline-NO cycle [6]. Additionally, L-citrulline is not subject to extensive pre-systemic degradation and has been shown to increase plasma L-arginine levels and endurance performance outcomes more efficiently than other NO-related supplements [7]. One study in healthy, middle-aged individuals demonstrated oral L-citrulline administration increased L-arginine plasma concentration and nitrate excretion, which were significantly correlated to flow-mediated vasodilation; this suggests L-citrulline-induced changes in plasma L-arginine may augment bioactivity of NO [7]. Furthermore, L-citrulline may modulate plasma nitrate concentrations, which in turn may lead to a reduction in oxygen costs for energy production during exercise as a result of improved blood flow and mitochondrial efficiency [8,9]. However, research in healthy, athletic populations investigating the efficacy of L-citrulline supplementation for NO precursors and vascular function is limited.

Nitric oxide and nitrates have been the focus of most research on vascular function and performance as they have consistently demonstrated the ability to increase blood flow and prolong time to exhaustion (TTE) [10,11]. In healthy, recreationally active adults, research on acute pomegranate juice supplementation, a rich source of nitrates, found significant increases in blood flow and vessel diameter during high-intensity exercise, which subsequently improved running TTE [12,13]. A large contributing factor to these vascular and performance improvements may be due to antioxidants found in pomegranate juice given the role of antioxidants in protection against free radicals. Glutathione (GSH) is an antioxidant that may modulate NO synthesis. Additionally, NO can react with GSH to form S-nitrosoglutathione, which is believed to stabilize NO, thereby protecting it from oxidative damage and possibly enhancing NO’s ergogenic effects [2,14]. Data evaluating acute administration of GSH paired with L-citrulline supplementation indicate that a synergistic relationship between GSH and NO exists [15]. Specifically, L-citrulline combined with GSH supplementation has been shown to increase plasma NO concentrations [15], which resulted in improved strength during resistance training [16]; this combination has yet to be evaluated in an aerobic exercise environment. During intense aerobic exercise, free radical production increases and may inhibit muscular contractile function, leading to muscle fatigue and decrements in performance [17]. L-citrulline combined with GSH supplementation may reduce the production of free radicals, thereby attenuating the negative impact of free radical formation during exercise and improving aerobic exercise performance.

While L-citrulline combined with GSH is known to enhance the effectiveness of resistance training, it is unclear whether it improves endurance performance. The purpose of the current study was to investigate the effects of L-citrulline + GSH on aerobic exercise performance and blood flow in well-trained men. The primary aim of this study was to determine the effects of orally delivered L-citrulline + GSH (Setria Performance Blend: SPB) vs. placebo on endurance performance, and blood flow before and after exercise in aerobically fit men. We hypothesized that the SPB would improve aerobic performance, increase blood flow, and increase vessel diameter compared the placebo. The secondary aim was to identify the effects of L-citrulline + GSH supplementation on blood metabolites of L-arginine, and L-citrulline. We hypothesized that the SPB would increase plasma concentrations of L-arginine and L-citrulline compared to the placebo.

## 2. Materials and methods

### 2.1. Subjects

**Figure 1.**
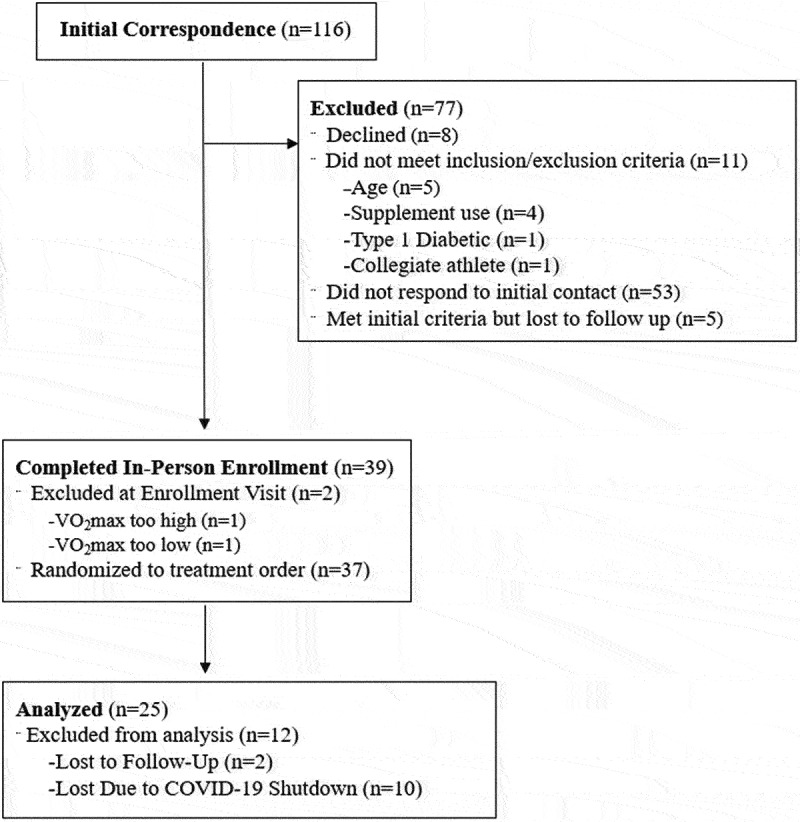
CONSORT recruitment. The five individuals who were active in the study during the mandatory stay at home orders issued in March 2019 in response to COVID-19 were stopped due to the university halt in research, not due to illness.

Twenty-five highly active males (Mean ± SD; Age: 22.2 ± 2.4 yrs; Height: 177.0 ± 4.8 cm; Weight: 75.3 ± 6.9 kg; VO_2_max: 50.5 ± 14.6 ml·kg^−1^·min^−1^) (Table 1) were recruited to participate in this study. Full CONSORT information is reported in Figure 1. All participants were healthy, active nonsmokers and were not consistently consuming any prescription drugs or supplements that would influence study outcomes such as creatine, beta-alanine, antihypertensive medications, whey protein, or anabolic steroids. All participants were required to achieve a VO_2_max of 42–70 ml·kg^−1^·min^−1^ prior to inclusion in the study, which classified their aerobic capacity as above average but below elite [18]. All methodology was approved by the University’s Institutional Review Board (IRB #19–1375), and all participants signed a written informed consent prior to participation.

**Table 1.** Anthropometric and descriptive characteristics of study participants (*n* = 25).

Age (yrs)	22.6 ± 2.4
Height (cm)	177.5 ± 4.6
Weight (kg)	75.3 ± 7.2
BMI (kg/m^2^)	23.9 ± 2.0
VO_2_max (mL/kg/min)	52.3 ± 6.2
Body Fat (%BF)	17.4 ± 4.2

Data are presented at mean ± standard deviation (SD).

### 2.2. Experimental design

**Figure 2.**
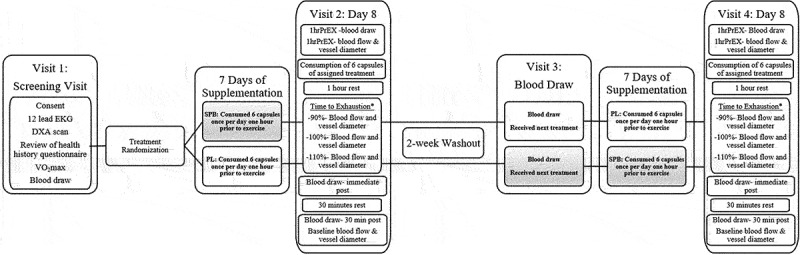
Experimental Design Figure [1hrPrEX- 1 hour prior to exercise; *time to exhaustion order was randomized per participant].

This study employed a randomized, double-blind, placebo-controlled, crossover design. Participants were asked to abstain from food and caloric beverages (12hrs), caffeine (12hrs), alcohol (24hrs), and physical activity (24hrs) prior to the baseline visit. Prior to the exercise testing sessions, participants were asked to abstain from food caloric beverages (4hrs), caffeine (12hrs), alcohol (24hrs), and physical activity (24hrs). Participants were instructed to refrain from using antibacterial mouthwash, chewing gum, and brushing their teeth on the morning of laboratory visits, as these factors may have modulated the conversion of nitrate to nitrite by influencing nitrate reductase enzymes in the oral cavity [19]. All participants completed a health history questionnaire to confirm inclusion/exclusion criteria, and underwent a 12-lead electrocardiogram (EKG) prior to baseline testing. Each participant completed a baseline maximal graded exercise test to volitional exhaustion to determine maximal oxygen consumption (VO_2_max) for inclusion and peak velocity (PV) to establish intensity for the high-intensity runs. Participants completed two separate exercise testing visits: one for the active treatment and one for the placebo. Both exercise testing visits consisted of three treadmill runs to exhaustion to determine blood flow, vessel diameter, and blood analytes. Blood flow was measured using a GE Logiq-e B-mode ultrasound (GE Healthcare, Wisconsin, USA) 60 minutes prior to exercise, immediately post each run to exhaustion, and 30 minutes post exercise. A minimum of fourteen days separated each testing visit, allowing for adequate washout between treatments (Figure 2).

### 2.3. Maximal oxygen consumption (VO_2_max)

To determine VO_2_max and peak velocity (PV), all participants completed a graded exercise test to volitional exhaustion on a treadmill. The treadmill was set at 10 km/h for the initial stage and increased by 2 km/h every 2 minutes until 16 km/h. At 16 km/h the stages increased 1k m/h every minute until 18 km/h, upon which percent grade was increased by 2% every minute. Breath-by-breath respiratory gases were analyzed with 15-second averages using indirect calorimetry (Parvo Medics TrueMax 2400®, Salt Lake City, UT); the three highest oxygen consumption values were averaged and recorded as VO_2_max (ml·kg^−1^·min^−1^). The test was considered maximal if it met a minimum of two of the following criteria: a plateau in heart rate (HR) or was within 10% of age-predicted HRmax; a plateau in VO_2_; a respiratory exchange ratio value greater than 1.15 [20]. Previous test-retest reliability for the protocol has demonstrated an intraclass correlation coefficient (ICC) = 0.99 and a standard error of measurement (SEM) = 1.59 ml·kg^−1^·min^−1^.

### 2.4. Body composition

Body composition was assessed via whole-body dual-energy x-ray absorptiometry scans (DXA; GE Lunar iDXA, GE Medical Systems Ultrasound & Primary Care Diagnostics, Madison, WI, USA) to characterize the stature of the sample. Participants were placed in the center of the scanning table after removing shoes and any clothing or jewelry containing metal or hard plastic. Participants were instructed to minimize movement during the scan, which was used to estimate lean mass (LM), fat mass (FM), and body fat percentage (BF%). Regions-of-interest from the default software (enCORE Software version 16) were adjusted by a trained technician. DXA test-retest reliability from this laboratory for individuals of similar stature included an ICC = 0.98 and SEM = 0.85 kg for FM, ICC = 0.99 and SEM = 1.07 kg for LM, and ICC = 0.96 and SEM = 1.279% for %fat.

### 2.5. Supplementation

Participants were provided an opaque bottle in a randomized order with half of the participants initially assigned to the Setria Performance Blend [SPB; L-citrulline (2 g) + glutathione (200 mg); Kyowa Hakko Bio, Tokyo, Japan] and the other half a placebo (PL; 3.1 g cellulose). Participants ingested their assigned treatment seven consecutive days prior to the first exercise visit (visit 2) and took the eighth dose at the laboratory. After a 14-day washout period, another baseline blood draw as obtained (visit 3), and participants began ingestion of the alternative treatment prior to the second exercise visit (visit 4). Utilizing a random allocation software, participants were randomly assigned treatment order in blocks of four to ensure equally balanced groups. Treatments were packaged in separate opaque bottles and blinded as treatment A and treatment B by the sponsor; both treatments were identical in appearance and taste. Participants were instructed to ingest six capsules of the assigned treatment once per day one hour prior to exercise for seven days. On day eight, participants were instructed to bring the treatment to the exercise visit where the eighth dose was consumed an hour before the exercise test (visits 2 and 4). Treatment consumption was separated by a minimum of a fourteen-day washout period, based on a half-life of ≤8 hours for both nitrate and L-citrulline [7,21]. To evaluate compliance, the remaining capsules from each treatment were counted. Based on the percentage of prescribed supplement that was consumed, average adherence for SPB was 90% and average adherence for PL was 88%. The data of subjects whose capsule intake rate was 80 to 120% were used for analysis, but data of those who exceeded (*n* = 1) or fell below (*n* = 1) the range were excluded from the analysis.

### 2.6. High-speed running

During the two exercise testing visits (visits 2 and 4) following the week-long supplementation regimens, participants arrived at the lab, ingested the final dose of the appropriate supplement, and waited 60 minutes before completing three separate treadmill runs to volitional fatigue at 90%, 100%, and 110% of the peak treadmill speed that was determined by PV from the VO_2_max test during visit 1. The varying intensities were randomly ordered for each participant but were maintained between both testing sessions per participant. Two recovery periods were provided between trial 1 and 2 and trial 2 and 3. During the recovery periods, HR was monitored, and participants were allowed to rest as long as it took for HR to return within 10% of resting HR or to reach a maximum rest period of 15  minutes. Time to exhaustion (TTE) was used to measure endurance (in seconds) for each run.

### 2.7. Brachial blood flow

Brightness-mode ultrasound (Logiq-e, GE Healthcare, Chicago, IL) was used to assess vessel diameter and blood flow through the brachial artery as previously described [12]. Measurements were taken on visits 2 and 4 one hour prior to exercise (1hrPrEX), immediately after each run to exhaustion (90%, 110%, and 110%), and 30 minutes post-exercise (30minPEX). For the 1hrPrEX and 30minPEX measure, a blood pressure cuff was positioned on the arm and inflated to 120 mmHg for 2 minutes then released immediately prior to measurement. For all measures, the participants were lying down with their right arm extended and positioned about 80 degrees away from the torso. The ultrasound was set to view continuous blood volume flow in the vascular, pulse wave, and colorflow setting. Transmission gel was applied to the ultrasound transducer probe (12LRS, 5–13 mhz), and the probe was held against the skin with sufficient pressure to obtain a clear image of the brachial artery without compressing its diameter. The ultrasound recorded a minimum of four pulses using the pulse wave setting. Artery diameter and arterial blood flow (aBF) were then estimated using the measure function in the device’s default software, similarly to the methods previously described by our research group for the brachial artery [12]. Test-retest reliability using these methods for brachial artery diameter from our laboratory include ICC = 0.82, SEM = 0.03 cm and blood flow ICC = 0.86, SEM = 5.92 mL∙min^−1^.

### 2.8. Blood analytes

A 5 ml venous blood sample was obtained from the antecubital region of the arm at visit 1, prior to VO2max, midpoint of the study (visit 3), and during visits 2 and 4 1hrPrEX, immediately post exercise (immediate PEX), and 30minPEX using one SST tube for each blood draw (Figure 2). Blood samples were allowed to coagulate at room temperature for 30  minutes, and then were immediately centrifuged at 3000 rpm at 4 °C for 10  minutes. Aliquots of serum were frozen at −80°C for batch analysis at the UNC Biobehavioral lab and Kotobiken, Medical Laboratories, Japan. Blood was sampled to evaluate the effects of supplementation on arginine, and L-citrulline. Commercially available, enzyme-linked assays were used to quantify arginine (Xevo TQ-S micro, LC-MS/MS, Kairos Waters Corporation, Millford, MA), and L-citrulline (Xevo TQ-S micro, LC-MS/MS, Kairos Waters Corporation, Millford, MA). All assays were performed in duplicate and averaged for analysis. Assay coefficients of variation ranged from 0.04 to 17%.

### 2.9. Statistical analyses

If a participant was missing data for two or more visits, they were excluded from the initial analysis. The data of subjects whose capsule intake rate was 80 to 120% were used for analysis, but data of those who exceeded or fell below the range were excluded from the initial analysis (*n* = 2). Multiple imputation was used to determine missing data values or outlier data values for compliant participants (*n* = 23). Outlier data points were removed if the value was 3 standard deviations (SD) above or below the mean [22]. A total of 25 data points were removed, which was less than one data point per time point (*n* = 42 timepoints).

Time to exhaustion values were evaluated using a series of separate repeated measures analyses of variance (ANOVAs) for treatment (SBP vs. PL) by TTE bout (90%, 100%, and 110%). Blood flow and vessel diameter variables were evaluated using separate repeated measures ANOVAs for each time point (1hrPrEX, 90%, 100%, 110%, and 30minPEX) between treatments; blood analyte variables were evaluated using a similar approach. Analyses were performed using SPSS (Version 26.0; IBM, Somers, NY, USA) with statistical significance set *a priori* at α = 0.05.

## 3. Results

Demographic and body composition values are presented in Table 1.

### 3.1. High-speed running

**Figure 3.**
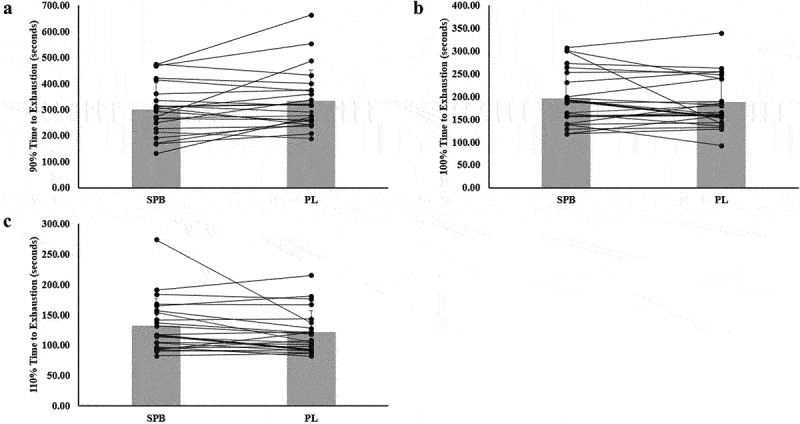
Individual responses and mean for vessel diameter (A-C) responses for compliant participants (*n* = 23). The lines represent the differences between SPB and PL per participant. The gray bars represent treatment mean with average standard deviation.

There was no significant treatment effect on TTE at 90% [Mean Difference (MD; SPB-PL) ± Standard Error (SE): −32.6 ± 16.3 sec; *p* = 0.058], 100% (MD: 8.1 ± 9.5 sec; *p* = 0.405), or 110% (MD: 10.4 ± 7.1; *p* = 0.159) (Table 2). Individual responses for TTE are presented in Figure 3.

**Table 2.** High-speed running data per treatment group.

%	SPB TTE (sec)	PL TTE (sec)	MD TTE (sec)	p-value
90	301.3 ± 100.8	334.0 ± 117.2	−32.6 ± 16.3	0.058
100	196.4 ± 62.2	188.3 ± 59.1	8.1 ± 9.5	0.405
110	132.0 ± 45.6	121.6 ± 35.5	10.4 ± 7.1	0.159

Data are presented as mean ± standard error (SE) and mean difference (MD) ± SE. SPB= active; PL= placebo; sec = seconds; TTE = time to exhaustion.

### 3.2. BrachiaL vessel diameter and blood flow

#### 3.2.1. Vessel diameter

Table 3 presents change in vessel diameter and blood flow after ingestion of the SPB and PL. There was no significant treatment effect on vessel diameter at 1hrPrEX (*p* = 0.980), 90% (*p* = 0.501), 100% (*p* = 0.747), 110% (*p* = 0.374), or at 30minPEX (*p* = 0.124).

**Table 3.** Comparison of vessel diameter and blood flows per treatment group.

	Vessel Diameter			
	SPB Diameter (cm)	PL Diameter (cm)	MD Diameter (cm)	p-value
1hrPrEX	0.43 ± 0.07	0.43 ± 0.04	0.000 ± 0.017	0.980
90%	0.41 ± 0.08	0.40 ± 0.05	0.011 ± 0.16	0.501
100%	0.40 ± 0.07	0.39 ± 0.04	0.003 ± 0.010	0.747
110%	0.41 ± 0.06	0.40 ± 0.06	0.014 ± 0.016	0.374
30minPEX	0.44 ± 0.06	0.42 ± 0.05	0.019 ± 0.012	0.124
	Blood Flow			
	SPB BF (ml/min)	PL BF (ml/min)	MD BF (ml/min)	p-value
1hrPrEX	109.6 ± 66.31	98.2 ± 49.5	11.5 ± 10.6	0.372
90%	109.5 ± 56.2	106.5 ± 35.4	3.0 ± 10.7	0.783
100%	90.0 ± 38.5	94.9 ± 35.9	−5.1 ± 10.6	0.631
110%	98.9 ± 57.0	87.7 ± 36.5	11.1 ± 13.9	0.433
30minPEX	186.4 ± 93.0*	127.0 ± 57.7*	59.4 ± 18.3*	0.004

SPB = active; PL = placebo; BF = blood flow; PrEX = prior to exercise; PEX = post exercise; MD = mean difference; *indicates significant difference between the treatments (*p* < 0.005).

#### 3.2.2. Blood flow

**Figure 4.**
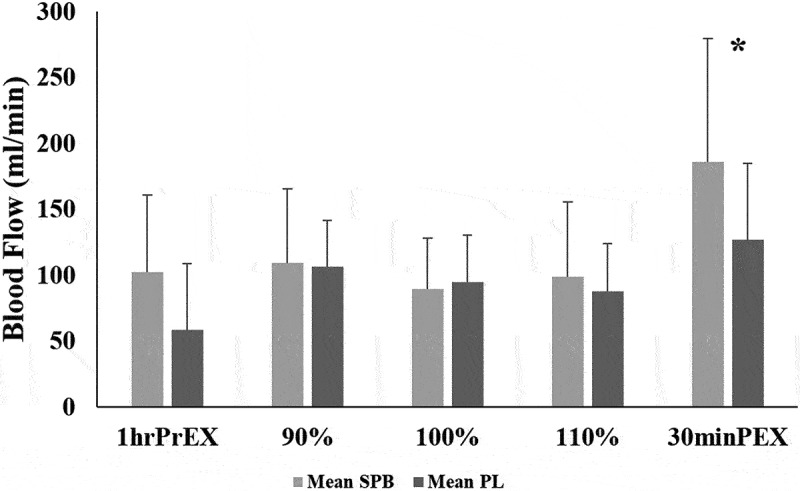
Mean ± standard deviation for blood flow across time points for compliant participants. *Indicates statistical significance between time points.

There was no significant treatment effect on blood flow at 1hrPrEX (*p* = 0.711), 90% (*p* = 0.783), 100% (*p* = 0.631), or 110% (*p* = 0.433). There was a significant treatment effect with SPB demonstrating significantly greater blood flow at 30minPEX exercise (*p* = 0.004) (Table 3; Figure 4).

### 3.3. Blood analytes

#### 3.3.1. Arginine

There was a significant difference between L-arginine concentration at baseline visits [(visit 1; 123.6 ± 22.5 nmol/L) vs. (visit 3; 134.8 ± 27.8 nmol/L; *p* = 0.050)]. There was no significant treatment effect on arginine concentration 1hrPrEX (*p* = 0.898); there was a significant treatment effect with SPB demonstrating significantly greater arginine concentration immediate PEX (*p* = 0.001) and 30 min PEX (*p* = 0.001) (Table 4).

**Table 4.** Comparison of blood analytes per treatment group.

	L-Arginine			
	SPB (nmol/L)	PL (nmol/L)	MD L-Arginine (nmol/L)	p-value
Visit 1 vs. Visit 3	123.6 ± 22.5*	134.8 ± 27.8*	−11.2 ± 5.4*	0.050
1hrPrEX	138.4 ± 26.2	137.6 ± 25.9	0.8 ± 6.0	0.898
Immediate PEX	150.2 ± 19.1*	119.8 ± 12.9*	30.4 ± 3.9*	0.001
30minPEX	141.5 ± 21.2*	116.6 ± 20.3*	24.9 ± 5.4*	0.001
	L-Citrulline			
	SPB (nmol/L)	PL (nmol/L)	MD L-Citrulline (nmol/L)	p-value
Visit 1 vs. Visit3	33.9 ± 8.5	33.8 ± 7.7	0.1 ± 1.3	0.916
1hrPrEX	35.4 ± 7.3	33.8 ± 8.9	1.6 ± 1.5	0.312
Immediate PEX	139.5 ± 34.8*	28.6 ± 6.4*	111.0 ± 7.4*	0.001
30minPEX	132.9 ± 28.8*	28.7 ± 7.4*	104.1 ± 11.2*	0.001

PrEX = prior to exercise; PEX = post exercise; MD = mean difference; *indicates significant difference between treatments (*p* < 0.05).

#### 3.3.2. L-citrulline

There was no significant difference between L-citrulline concentration at baseline visits [(visit 1; 33.9 ± 8.5 nmol/L) vs. (visit 3; 33.8 ± 7.7 nmol/L; *p* = 0.916)]. There was no significant treatment effect on citrulline concentration 1hrPrEX (*p* = 0.312); there was a significant treatment effect on citrulline concentration with SPB demonstrating significantly greater immediate PEX (*p* = 0.001) and 30-min PEX (*p* = 0.001) (Table 4).

## 4. Discussion

Endurance performance largely depends on increased oxygenation and blood flow to skeletal muscle [23]. Arginine and citrulline-based supplements may enhance the production of NO, thereby mediating smooth muscle relaxation and promoting vasodilation, greater blood flow regulation, and improvements in oxygen delivery and mitochondrial respiration [24–27]. While not significant, vessel diameter was augmented at the 90% and 110% intensities, and 30 minutes post-exercise following ingestion of SPB. Compared to PL, acute ingestion of SPB resulted in a significant increase in blood flow 30 minutes post-exercise. Previous research has demonstrated that oral L-citrulline supplementation is effective for increasing L-arginine [7], as well as various biomarkers of NO [15]. The present study demonstrated significant increases in L-arginine and L-citrulline.

NO-related supplements are growing in popularity; however, to date there is a lack of consensus regarding the effects of L-citrulline and L-arginine on aerobic exercise performance [6]. Citrulline supplementation has been evaluated with various methodologies differing in dosing and aerobic performance outcomes. Existing studies have utilized a range of~2.4 to 6 grams per day of citrulline for up to seven days, followed by evaluation of performance via time trials or VO_2_max testing [28,29]. Oral L-citrulline supplementation has previously resulted in increases in plasma L-arginine and NO concentration [26], indirectly resulting in increased exercise performance [30], tolerance to high-intensity exercise [31], blood flow [5], and reductions in lactate and ammonium accumulation [8,32]. While some studies have demonstrated increases in plasma L-arginine and aerobic exercise performance with L-citrulline supplementation in recreationally trained individuals [26,28,29,31]. other studies have not [33–35]. It remains unclear whether supplementation may enhance aerobic performance in trained individuals, despite positive results of L-citrulline supplementation in recreationally trained individuals, particularly when considering differences in endothelial and mitochondrial adaptations. In the present study, acute supplementation with SPB did not have an ergogenic effect on TTE. Similar to our findings, one study observing the effects of ~3.4 grams of L-citrulline supplementation per day over 16 days in eight healthy, aerobically trained males demonstrated no significant differences in TTE between the placebo or supplement group despite significant increases in plasma arginine [34]. Furthermore, the magnitude of improvement in TTE between treatment types in the present study may have been limited by adaptations to high-intensity exercise since the participants in this study were aerobically trained individuals. However, previous research has suggested a ~ 20% improvement in TTE may correspond to a ~ 1–2% improvement in exercise performance, which could have profound impacts at high levels of competition [36]. While not significant, SPB supplementation augmented TTE by ~10  seconds at 110% intensity, signifying L-citrulline supplementation may have positive effects on high-intensity exercise performance in aerobically trained males.

Due to the role of NO in vasodilation and improvements in blood circulation, many studies have investigated the mechanisms of NO-related supplements on aerobic exercise performance [10,11]. Vasodilation from NO may aid in the delivery of nutrients and oxygen to the working muscles during exercise thereby enhancing performance and supporting quicker recovery. L-arginine is the direct precursor to NO in the nitric oxide synthase (NOS)-dependent pathway suggesting increases in plasma L-arginine may result in enhanced NO production augmenting positive endothelial effects during exercise [31,37]. L-arginine plasma concentrations have improved as a result of L-citrulline supplementation [7,38]. Therefore, it is possible that L-citrulline supplementation also enhances vasodilation, particularly through increasing plasma levels of L-arginine and NO bioavailability. While the present study demonstrated significant increases in L-arginine levels ImmediatePEX (MD: 30.4 nmol/L) and 30minPEX (MD: 24.9 nmol/L) with SPB supplementation, there were no significant increases in vessel diameter across exercise intensities or between groups. The SPB group had significantly lower baseline levels of L-arginine, which may have impacted the magnitude of vasodilation despite the significant increase in L-arginine concentration with exercise. However, the difference between the baseline measures was not meaningful beyond the error of measurement for the assay, suggesting the significance between visit 1 and visit 3 may have been due to variability in the assay. A previous investigation evaluating pomegranate extract supplementation, which is rich in nitrates, in highly active males and females utilizing a similar exercise protocol to the present study, demonstrated significant increases in vessel diameter 30 minutes post-exercise compared to a placebo [12]. The authors suggested that in addition to the nitrates, the antioxidants found in pomegranate extract may have augmented increases in flow-mediated vasodilation, thereby enhancing the endothelial NOS expression [12,39]. Previous research has demonstrated a strong mechanistic basis underlying the synergistic relationship between antioxidants, such as GSH as used in the present study, and L-citrulline. Due to its effects on NOS, GSH may protect against the oxidative reduction of NO [7]. The addition of an antioxidant source to L-citrulline supplementation may produce a positive effect on the ergogenic capacity, particularly in aerobically trained individuals [2,40].

In addition to vasodilation, NO promotes increases in blood flow enhancing nutrient, hormone, and oxygen delivery to exercising muscles [6,41]. L-arginine has been a primary ingredient in most NO-stimulating dietary supplements due to the proposed benefit of increased blood flow to the myocardium and skeletal muscle [27]. However, most of the previous research demonstrating positive effects of L-arginine supplementation on blood flow occurs in subjects with cardiovascular or pulmonary diseases [27,42–44]. In healthy individuals, L-arginine supplementation has demonstrated limited efficacy on exercise performance and NO synthesis due to significant catabolism due to the enzyme arginase, which hydrolyzes L-arginine to L-ornithine and urea resulting in low plasma L-arginine levels [6,26,45–47]. Unlike L-arginine, L-citrulline catabolism is limited to the intestines, resulting in the majority of L-citrulline passing into systemic circulation before conversion to L-arginine [48,49]. As such, L-citrulline supplementation may be more effective for increasing L-arginine bioavailability for subsequent NO production [7] (for a comprehensive review of the metabolic pathways, see Jones et al. [50]). One study reported that three grams of L-citrulline supplementation per day for seven days in 20 healthy adults resulted in increased plasma L-arginine concentrations, and urinary cyclic guanosine monophosphate and nitrate excretion, ultimately indicating enhanced NO production and bioactivity [7]. Furthermore, there was a strong positive correlation between the change in flow-mediated vasodilation pre- and post-supplementation (*r* = 0.92, *p* = 0.001), suggesting that the L-citrulline-induced changes in plasma L-arginine translated to meaningful effects on the bioactivity of NO [7]. Our results support previous research demonstrating enhanced blood flow 30minPEX for SPB ingestion compared with placebo (mean difference ± standard error: 59.4 ± 18.3 ml/min; *p* = 0.004). Enhanced blood flow 30minPEX supports an ergogenic effect of L-citrulline and potential implications for exercise recovery. The hyperemic response to exercise may have concealed any significant changes to blood flow or vessel diameter in the measurements taken immediately after each run to exhaustion and led to the inconsistent increases in blood flow and vessel diameter at each time point. Previous research has suggested that during intense whole-body exercise, such as high-intensity treadmill running, the body prioritizes increasing blood flow to the working muscles prior to maximizing vessel diameter [51]. Furthermore, the physiological limits of vessel compliance and intact regulatory mechanisms in healthy, active individuals may limit the effects of L-citrulline supplementation on blood flow in more aerobically trained individuals [52]. Indeed, other studies utilizing L-citrulline paired with high-intensity resistance training have demonstrated increases in NO concentrations [16,53], yet these did not evaluate blood flow during the exercise session.

Nitrates and nitrites have consistently been used as indicators of NO status, as a result of the rapid metabolism of L-arginine and L-citrulline [46]. L-citrulline, when combined with GSH, has demonstrated greater increases in plasma NO markers than L-citrulline alone [15,54]. The present study supports improvements in plasma L-citrulline and L-arginine after eight days of L-citrulline + GSH supplementation. L-citrulline and L-arginine plasma concentrations were significantly elevated immediately PEX (MD: 111.0 and 30.4 nmol/L, respectively) and 30minPEX (MD: 104.1 and 24.9 nmol/L, respectively) following SPB supplementation when compared to placebo.

Previous research in recreationally trained individuals has demonstrated that L-citrulline supplementation may improve aerobic performance markers such as TTE and aerobic capacity [26]. The present study targeted aerobically trained individuals (VO_2_max: 50.5 ± 14.6 ml·kg^−1^·min^−1^). Despite positive results in other populations, it remains unclear whether L-citrulline supplementation may enhance performance in more trained aerobic athletes. Future research may benefit from evaluating the effects of L-citrulline between aerobic athletes and recreationally active individuals to assess the impact of training status on performance and blood analyte outcomes. A limitation of the present study is that the high-intensity exercise is a stimulus for blood flow alone, thereby possibly convoluting the results surrounding blood flow and vessel diameter. However, the study design utilized in the present study previously demonstrated significant increases in vessel diameter, blood flow, and delayed fatigue in highly active participants supplementing with pomegranate extract [12]. Additionally, another possible limitation is the present study utilized only 2 grams of L-citrulline+200 milligrams of GSH over 7 days, a dose that has previously demonstrated significant improvements in nitrate concentration after a bout resistance training [15]. While this dose may be beneficial in anaerobic exercise, it is possible that a higher dose of L-citrulline (~6 grams per day) is needed to observe meaningful differences in aerobic exercise. Future research should consider investigating if a higher dose per day of L-citrulline + GSH has a more profound impact on performance, blood flow, and biomarkers of NO metabolism.

## 5. Conclusion

In conclusion, L-citrulline + GSH supplementation did not enhance TTE in aerobically trained men. Although SPB supplementation did not translate to improved performance, there was a significant increase in blood flow at 30 minutes following exercise. Increased blood flow after exercise may promote exercise recovery due to augmented nutrient, hormone, and oxygen delivery. In agreement with previous reports of increased L-arginine from oral L-citrulline supplementation [7], there was a meaningful change in blood markers of L-arginine and L-citrulline. Further research is needed to elucidate potential mechanisms related to L-citrulline supplementation in other aspects of performance.
